# Prediction of the Risk Distributions for *Anopheles sinensis*, a Vector for Malaria in Shanghai, China

**DOI:** 10.4269/ajtmh.22-0523

**Published:** 2023-01-23

**Authors:** Yi-xin Tong, Zhi-gui Xia, Qiao-yan Wang, Ning Xu, Hong-lin Jiang, Zheng-zhong Wang, Ying Xiong, Jiang-fan Yin, Jun-hui Huang, Feng Jiang, Yue Chen, Qing-Wu Jiang, Yi-Biao Zhou

**Affiliations:** ^1^ Fudan University School of Public Health, Shanghai, China;; ^2^ Key Laboratory of Public Health Safety, Fudan University, Ministry of Education, Shanghai, China;; ^3^ Fudan University Center for Tropical Disease Research, Shanghai, China;; ^4^ National Institute of Parasitic Diseases, Chinese Center for Disease Control and Prevention, Shanghai, China;; ^5^ Jiading District Center for Disease Control and Prevention, Shanghai, China;; ^6^ School of Epidemiology and Public Health, Faculty of Medicine, University of Ottawa, Ottawa, Canada

## Abstract

Malaria is a parasitic disease caused by *Plasmodium*, and *Anopheles sinensis* is a vector of malaria. Although malaria is no longer indigenous to China, a high risk remains for local transmission of imported malaria. This study aimed to identify the risk distribution of vector *An. sinensis* and malaria transmission. Using data collected from routine monitoring in Shanghai from 2010 to 2020, online databases for *An. sinensis* and malaria, and environmental variables including climate, geography, vegetation, and hosts, we constructed 10 algorithms and developed ensemble models. The ensemble models combining multiple algorithms (*An. sinensis*: area under the curve [AUC] = 0.981, kappa = 0.920; malaria: AUC = 0.959, kappa = 0.800), with the best out-of-sample performance, were used to identify important environmental predictors for the risk distributions of *An. sinensis* and malaria transmission. For *An. sinensis*, the most important predictor in the ensemble model was moisture index, which reflected degree of wetness; the risk of *An. sinensis* decreased with higher degrees of wetness. For malaria transmission, the most important predictor in the ensemble model was the normalized differential vegetation index, which reflected vegetation cover; the risk of malaria transmission decreased with more vegetation cover. Risk levels for *An. sinensis* and malaria transmission for each district of Shanghai were presented; however, there was a mismatch between the risk classification maps of *An. sinensis* and malaria transmission. Facing the challenge of malaria transmission in Shanghai, in addition to precise *An. sinensis* monitoring in risk areas of malaria transmission, malaria surveillance should occur even in low-risk areas for *An. sinensis*.

## INTRODUCTION

Malaria is a parasitic disease caused by *Plasmodium*.^1^ As the WHO reported in 2020, there were ∼229 million malaria cases and 409,000 related deaths in 87 countries.^2^ Although great strides have been made in global malaria control,^2^ malaria risk areas have not changed significantly over the past half-century.^3^ On the basis of previous research, 2.5 billion people are at risk for *Plasmodium vivax* malaria worldwide,^4^ whereas 1.13 and 1.44 billion people in the world are at risk for unstable and stable *Plasmodium falciparum* malaria, respectively.^5^ In particular, the retransmission of malaria resulting from imported cases has been reported in several countries, such as the United States, South Korea, and Italy, after the announcement of malaria elimination,^6^ raising public concerns about the possible resurgence of the disease.^7^^,^^8^ Related studies have been implemented in other southern European countries, and the results have shown that the resurgence risk is high.^9^^–^^11^

China was once a major endemic area for malaria. The country has achieved great success in the prevention and control of the disease with a WHO certification of malaria elimination issued in June 2021.^12^^,^^13^ However, imported cases occur in all provinces,^14^^–^^16^ and the number of imported malaria cases has shown an increasing trend year by year.^16^ An analysis of imported malaria in southeastern China indicates that the proportion of falciparum malaria leading to severe outcomes reached 76.3% (765/1,003) of the total malaria cases during the period 2012–2016, which is the predominant malaria with a serious health threat to the population.^17^ The vector *Anopheles sinensis* is widely distributed,^18^ and the population is generally susceptible to *Plasmodium*.^19^ In other words, a complete malaria transmission chain still exists that causes secondary transmission in China.^20^ Thus, how to identify hot spots for malaria reemergence in focus areas and develop a generic model for retransmitted malaria has gradually become the key and future focus of malaria prevention in China.^21^^,^^22^

Variability and transmission of malaria are greatly influenced by environmental changes.^18^^,^^23^^,^^24^ Multiple variables, including precipitation, temperature, altitude, and population, can affect the transmission process of malaria through sources of infection, transmission routes, and susceptible individuals.^3^^,^^25^ To evaluate the risk of malaria transmission in central Italy, Romi et al. combined a multifactorial approach with climatic parameters.^6^ The findings from Lee et al. demonstrated an increased risk of imported malaria in Asian-born populations in Minnesota.^26^ Previous studies also showed that vector *An. sinensis* spatial distribution was associated with malaria risk distribution.^27^^–^^29^ However, few studies were conducted to identify the broader environmental conditions for vector *Anopheles* and malaria transmission after the elimination of malaria.^24^^,^^29^^,^^30^

Applying various methods to model the spatial distribution of vector species can assist in the assessment and management of associated health risks.^31^ Studies using different modeling techniques to quantify the mathematical and physical relationships between diseases and the environment for the prediction of possible distribution are relatively widespread.^32^^–^^35^ Although both linear^36^ and nonlinear models^3^^,^^18^^,^^37^^,^^38^ have been used in previous studies, Song et al. observed that generalized addictive models performed better than linear models in spatial distribution estimation for malaria.^3^

Species distribution models are pivotal tools for forecasting and comprehending species distributions and have successfully identified the risk distribution of a disease in an area by changing environmental variables, including a dozen algorithms.^39^^–^^41^ Messina et al. used the boosted regression tree to predict the distribution of dengue and population at risk.^42^ Manyangadzer et al. used the negative binomial generalized linear mixed model to predict the spatial distribution of schistosomiasis infections.^43^ In addition, an ensemble forecast technique has been used to account for the variability among various algorithms to obtain the central tendency.^44^ The ensemble model combines several modeling approaches into a single predictive model to decrease variance and bias and improve prediction considerably.^45^^–^^47^

Shanghai is a high-risk area for malaria transmission. In this study, we focused on the spatial risk of the local malaria transmission resulted from the imported malaria patients as the source of infection. We constructed 10 algorithms and developed ensemble models to determine hierarchically the risk distributions for vector *An. sinensis* and malaria transmission and their environmental predictors. We also examined the association of risk classes between *An. sinensis* and malaria transmission risk classifications. The results of our study will help carry out precise prevention and consolidate the achievements of malaria control to meet the requirements of the WHO Global Vector Control Response (2017–2030).^2^

## MATERIALS AND METHODS

### Study area.

Shanghai was an endemic area for malaria with an annual incidence rate of more than 3% in the 1950s.^14^ Located in the Yangtze River Delta in eastern China between 120°52′ and 122°12′ E longitude and 30°40′ and 31°53′ N latitude, Shanghai currently has approximately 26 million people living within 63.405 million km^2^ and is one of the most densely populated areas in the world. It has a subtropical monsoon climate with a mild and humid environment, which is suitable for *Anopheles* breeding.^48^ The region has increasing trends of trade exchanges, high population density, and imported malaria cases.

### Occurrence data.

We conducted an extensive literature search by using the keywords “*Anopheles sinensis*,” and the full text included “Shanghai” in China National Knowledge Infrastructure, Wanfang Database, and Weipu Database between 2010 and 2020. Review and experimental articles were excluded. A comprehensive literature review containing the title of the literature, the species of *An. sinensis*, and the occurrence records with time and location for *An. sinensis* was conducted. We collected all occurrence records in Shanghai from the comprehensive literature review, Global Biodiversity Information Facility database (GBIF, http://www.gbif.org/), and occurrences of *An. sinensis* found in normative field surveillance conducted by Jiading District Center for Disease Control and Prevention in the Jiading District of Shanghai in 2020. Then, a raw database containing occurrence records for *An. sinensis* was formed. Data from 451 malaria cases in Shanghai from 2010 to 2020 extracted from the Chinese National Disease Surveillance Reporting System were used to build the raw database for malaria.

**Figure 1.**
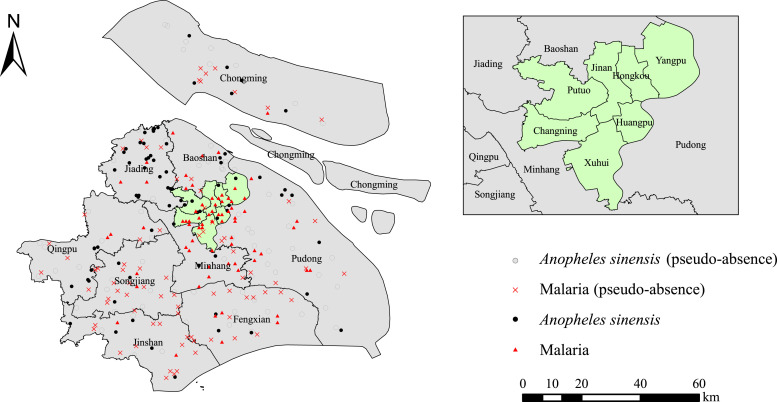
Geographic distributions of *Anopheles sinensis* and malaria cases.

We extracted all available location information for each occurrence in the two raw databases. Google Earth (https://www.google.com/earth/) was used to acquire the coordinates of collection points when the information was not provided. The occurrences with missing geospatial information and absent environmental variable layers were excluded. Duplicate records were removed, and only one presence point per grid (1 km × 1 km) was retained to reduce spatial autocorrelation.^49^^–^^51^ Figure 1 shows the presence of malaria and *An. sinensis* for modeling in our study.

### Environmental variables.

We acquired climatic, geographic, and vegetation data including average annual temperature (AAT), average annual precipitation (AAP), ≥ 0°C annual accumulated temperature (AAT0DEM), ≥ 10°C annual accumulated temperature (AAT10DEM), aridity, moisture index (IM), elevation, aspect, normalized difference vegetation index (NDVI) from the Resource and Environmental Science Data Center of the Chinese Academy of Sciences (http://www.resdc.cn). We obtained host data, including human footprint index, human influence index, and night light index (NLI) through the International Center for Earth Science Information Network (http://sedac.ciesin.columbia.edu) and the Resource and Environmental Science Data Center of the Chinese Academy of Sciences (http://www.resdc.cn). The raster data of the preceding 12 environmental variables were unified into 1 km × 1 km resolution. In addition, we unified the extent of Shanghai in all maps by using ArcGIS 10.2.

**Figure 2.**
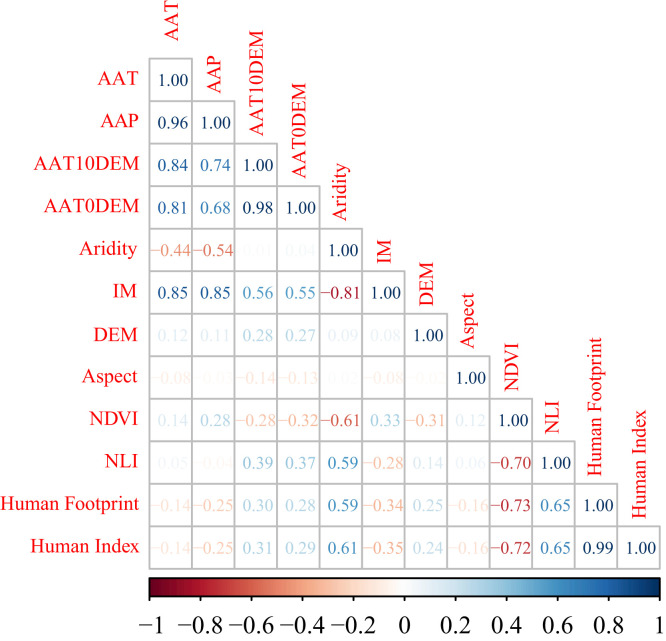
Pearson correlation analysis of variables. AAP = average annual precipitation; AAT = average annual temperature; AAT0DEM = ≥ 0°C annual accumulated temperature; AAT10DEM = ≥ 10°C annual accumulated temperature; DEM = elevation; IM = moisture index; NDVI = normalized difference vegetation index; NLI = night light index.

To avoid the effects of overparameterization and multicollinearity among variables,^47^ we calculated Pearson correlation coefficients among variables (Figure 2) and removed variables with high a correlation (*r* > 0.9).^34^ AAP, AAT0DEM, and Human Index were excluded. In addition to the nine environmental variables that were applied in the *An. sinensis* prediction model, the predicted risk distribution for *An. sinensis* was incorporated into the malaria prediction model (Table 1).

**Table 1:** Variables involved in model construction

Descriptor type	Abbreviation	Variable description	Period	Model type
Climate	AAT	Average annual temperature	2010–2015	*Anopheles sinensis*, malaria
	AAT10DEM	≥ 10°C annual accumulated temperature	1950–1990	*An. sinensis* malaria
	Aridity	Aridity	1950–1990	*An. sinensis*, malaria
	IM	Moisture index	1950–1990	*An. sinensis*, malaria
Geography	DEM	Elevation	2000	*An. sinensis*, malaria
	Aspect	Aspect	2000	*An. sinensis*, malaria
Vegetation	NDVI	Normalized differential vegetation index	2010–2020	*An. sinensis*, malaria
Host	Human Footprint	Human footprint index	1995–2004	*An. sinensis*, malaria
	NLI	Night light index	2000–2013	*An. sinensis*, malaria
Vector	ARD	*An. sinensis *risk distribution	2010–2020	Malaria

### Model development and assessment.

The biomod2 package of the R 4.1.0 was used to construct models with 10 algorithms: general linear models, general boosted models (GBM, also referred to as boosted regression trees), general additive models (GAM), classification tree analysis (CTA), artificial neural networks (ANN), surface range envelope (SRE), flexible discriminant analysis (FDA), multiple adaptive regression splines (MARS), random forests (RF), and maximum entropy (MAXENT).^52^ We introduced pseudo-missing records by 1:1 random sampling for some models^32^ that required information that was missing. Figure 1 shows the pseudo-absence points of malaria and *An. sinensis* for modeling in our study.

Data of all the points in the original datasets were randomly split into two parts, with 75% calibrating the model (training data) and 25% evaluating the model (testing data). To reduce overfitting, we repeated the process of dividing the data, calibrating, and evaluating the model 10 times and obtained 100 models in total (10 modeling algorithms × 10 repeated runs). Although the ratio of the testing and training data in the original datasets remained the same, the testing data and training data differed in each run. Receiver operating characteristic curve (area under the curve [AUC]) and the kappa statistic were used to evaluate and compare synthetically the out-of-sample performance of the models on the testing data. The ability of the model to distinguish between occupied and unoccupied sites was evaluated by AUC. Also, the kappa statistic was used to evaluate model consistency.^47^ When evaluating kappa, the models transformed the predictions into binary predictive maps with a threshold when the kappa value was maximal. A high value close to 1.0 reflects a strong signal of an excellent model performance.^53^ Models with AUC > 0.7 and kappa > 0.4 were weighted by AUC to develop ensemble models.^6^^,^^54^^–^^56^ We also calculated the ranks for the importance of environmental variables to compare the out-of-sample performance among models.

Limited by the small scale of Shanghai and by the spatial resolution of the variables, the predictive raster layers created by the ensemble models for *An. sinensis* and malaria transmission were composed of the 1 km × 1 km grids. To minimize grid-to-grid contact surface differences and make logical transitions to improve the resolution of raster layers for viewing, we used Kriging interpolation over the raster layers created by the ensemble models.^57^^,^^58^ Further, to compare the impact on the raster layers before and after Kriging interpolation, fuzzy kappa implemented in the Map Comparison Kit version 3.2.3 was used to compare these raster layers with unequal resolution per grid statistically.^59^ Between 0 (completely different maps) and 1 (identical maps), the fuzzy kappa indicates the average agreement between two maps compared with the expected agreement from random relocation of all grid squares in two maps.^59^^–^^61^ The values of fuzzy kappa after Kriging interpolation were 0.939 for *An. sinensis* mapping and 0.942 for malaria transmission mapping with excellent consistency, and the differences of all the grid squares caused by Kriging interpolation are shown in Supplemental Figure 1.

### Risk classification.

The ensemble models were ultimately selected to draw the risk classification maps of *An. sinensis* and malaria transmission. The final output predictions from the ensemble models had a range of values from 0 to 1. These values as the variable reflected the likelihood of occurrence, which were regrouped into four risk classes: no risk (0.00–0.40), low risk (0.41–0.60), intermediate risk (0.61–0.80), and high risk (0.81–1.00).

In addition, by using ArcGIS 10.2 to compare the consistency of risk classes for the output predictions of *An. sinensis* and malaria transmission per grid, we examined the risk class association by assessing whether the risk levels of the areas in both maps were the same.

## RESULTS

### Model out-of-sample performance.

**Figure 3.**
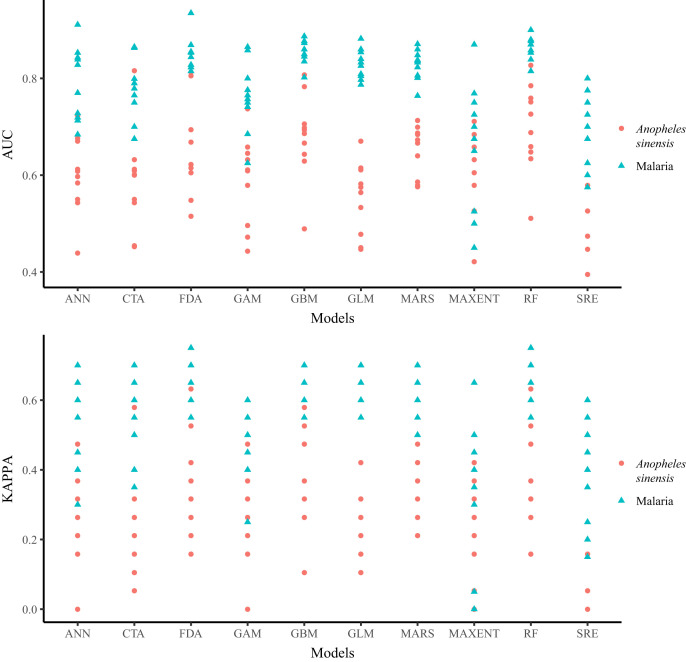
Out-of-sample performance in terms of area under the curve (AUC) and kappa over the 100 predictive models (10 algorithms × 10 repeated runs). ANN = artificial neural networks; CTA = classification tree analysis; FDA = flexible discriminant analysis; GAM = general additive models; GBM = general boosted models; GLM = general linear models; MARS = multiple adaptive regression splines; MAXENT = maximum entropy; RF = random forests; SRE = surface range envelope.

Figure 3 shows the out-of-sample performance of 10 algorithms. Random forests (*An. sinensis*: AUC = 0.827, kappa = 0.632) and FDA (AUC = 0.935, kappa = 0.750) for malaria had the best overall out-of-sample performance, and SRE (AUC = 0.395, kappa = 0.010) for *An. sinensis* and MAXENT (AUC = 0.450, kappa = 0.010) for malaria had the worst overall out-of-sample performance.

The *An. sinensis* ensemble model was obtained from 13 models with AUC > 0.7 and kappa > 0.4 (three models using RF or GBM algorithm, two models using FDA, and one model MAXENT, MARS, CTA, ANN, or GAM algorithm), and the malaria ensemble model was obtained from 85 models including all 10 algorithms. Compared with any single model, the out-of-sample performance of the ensemble models was best (*An. sinensis*: AUC = 0.981, kappa = 0.920 and malaria: AUC = 0.959, kappa = 0.800), and was applied to classification mapping.

### Importance analysis of environmental predictors.

**Figure 4.**
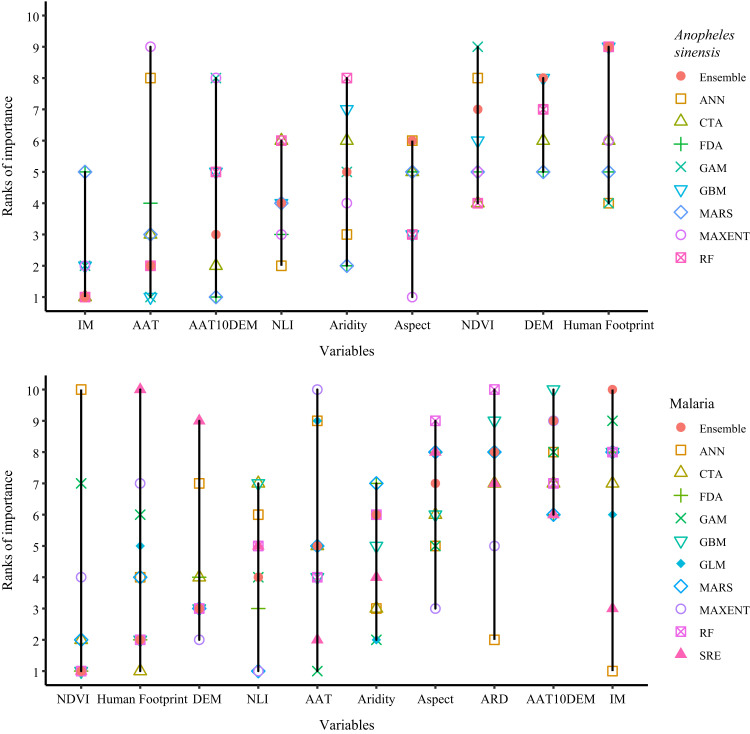
Ranking of importance of the contribution of each variable of different algorithms in the final ensemble models. The length of the vertical bar indicated the difference in the rank of importance for the same variable in the above algorithms. AAT = average annual temperature; AAT10DEM = ≥ 10°C annual accumulated temperature; ANN = artificial neural networks; ARD = *Anopheles sinensis* risk distribution; CTA = classification tree analysis; DEM = elevation; FDA = flexible discriminant analysis; GAM = general additive models; GBM = general boosted models; IM = moisture index; MARS = multiple adaptive regression splines; MAXENT = maximum entropy; NDVI = normalized difference vegetation index; NLI = night light index; RF = random forests; SRE = surface range envelope.

Figure 4 shows the ranked importance of environmental predictors of different algorithms in the final ensemble models. For *An. sinensis*, IM, as an indicator of the degree of wetness, was the most important environmental predictor in the ensemble model. The higher the degree of wetness, the lower the risk of *An. sinensis*. Average annual temperature and AAT10DEM, as indicators of average temperatures, also ranked high in some models.

For malaria transmission, NDVI, as an indicator of the vegetation cover status, showed the highest importance in the ensemble model; the risk of malaria transmission decreased with greater vegetation cover. NLI—that is, the brightness of night light, which reflects socioeconomic conditions—and Human Footprint, as the quantitative indicator of the impact of human activity, also ranked high in some models.

### Prediction of *An. sinensis* risk distribution in Shanghai.

**Figure 5.**
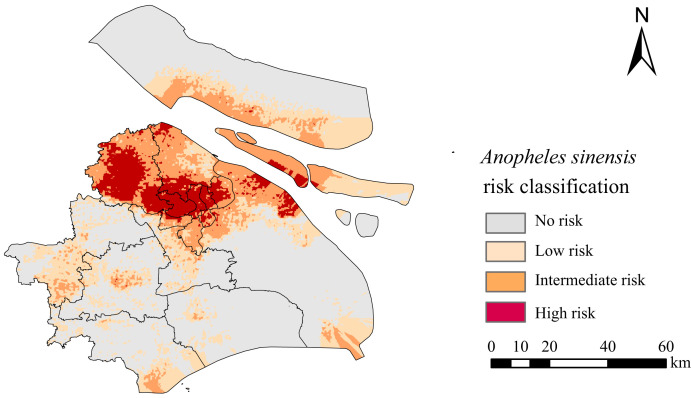
Classification map of *Anopheles sinensis* risk in Shanghai predicted by the ensemble model.

*Anopheles sinensis* risk areas were found in all districts of Shanghai, with high-risk areas concentrated in the northern part of the city (Figure 5). The high risk areas of *An. sinensis* were mainly located in the Jiading and Baoshan districts, showing a certain aggregation. Other districts, such as Putuo, Jingan, and Hongkou, also had a small number of high-risk areas for *An. sinensis*.

### Classification of malaria cases in Shanghai.

**Figure 6.**
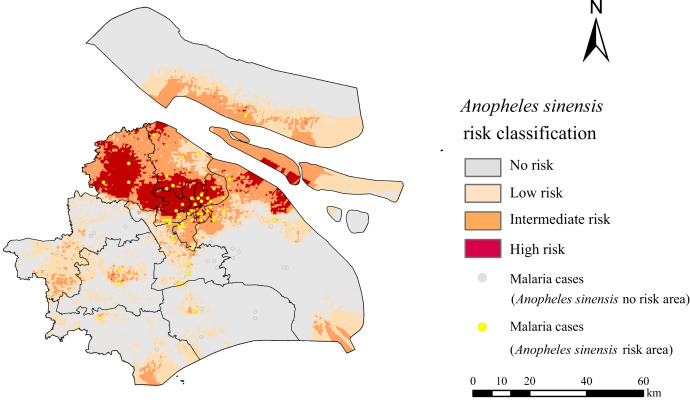
Classification of malaria cases in Shanghai based on the classification map of *Anopheles sinensis* risk.

Eighty unique spatial locations in Shanghai were identified from 451 malaria cases. The locations of reported malaria cases were distributed in all the 16 districts of Shanghai, mainly in central and northern Shanghai. As a result, 73.8% (59/80) of the reported malaria cases were located in *An. sinensis* risk areas, and 26.2% (21/80) in no risk areas of *An. sinensis* (Figure 6).

### Prediction of malaria transmission risk in Shanghai.

**Figure 7.**
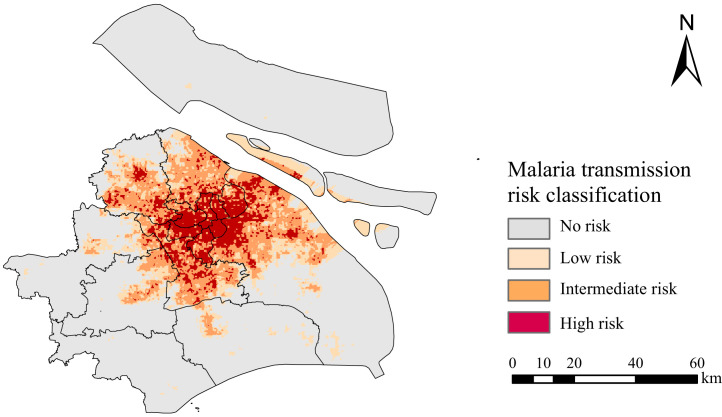
Classification map of malaria transmission risk in Shanghai predicted by the ensemble model.

Risk areas of malaria transmission were distributed in all the districts and mainly concentrated in central Shanghai (Figure 7). Among them, the Pudong district had the largest transmission risk area for malaria. The high-risk areas showed aggregation in the city center covering 11 districts. For example, Pudong had the largest high-risk area, followed by Putuo, Jingan, Hongkou, and other districts in the city center. In contrast, there were few high-risk areas in the far suburbs of the city.

### Risk classification mismatch between malaria transmission and *An. sinensis*.

**Figure 8.**
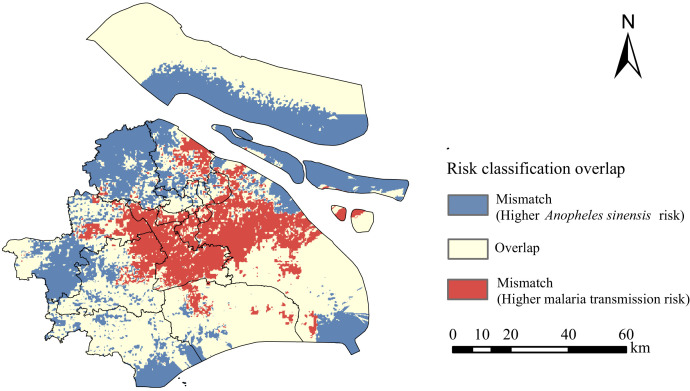
Map of the risk classification overlap between *Anopheles sinensis* and malaria.

There was a lack of strong risk class association between the risk distributions of malaria transmission and *An. sinensis* (Figure 8). Comparing the two predictive risk maps of malaria transmission and *An. sinensis*, the overlap of risk classification was only 3,410.9 km^2^ (53.8%). Among the areas with mismatched classification, the higher risk level for malaria transmission was 1,024.7 km^2^ (16.1%), and the higher risk level for *An. sinensis* was 1,908.4km^2^ (30.1%).

## DISCUSSION

The study provided hierarchical maps of risk distributions for the vector *An. sinensis* and malaria transmission predicted by ensemble models with multiple modeling algorithms. The risk areas of both *An. sinensis* and malaria transmission were found to be mainly concentrated in the north and central areas, which required attention. Currently, there are still some high-risk areas that lack routine surveillance for *An. sinensis*, such as the Chongming and Baoshan districts.

Only 73.4% (59/80) of reported malaria cases were located in *An. sinensis* risk areas (Figure 6), suggesting that vector monitoring at a finer scale is not enough. We compared the two predictive risk classification maps and found that the risk distributions of malaria transmission and *An. sinensis* did not correspond to each other (Figure 8). Similar results have been observed in Europe, indicating that autochthonous transmission is low despite the documented presence of *Anopheles*.^62^ A similar mismatch was also found for the risk distributions of Chagas disease and *T. infestans*.^63^ A possible reason is that for Shanghai, where the malaria elimination goal has been achieved, host factors, such as the sites of imported malaria cases and NLI, played a greater role than presence of the vector *An. sinensis*. This may partly explain the resurgence of malaria in other countries where mosquito surveillance is incomplete.^6^^,^^64^^–^^66^ The mismatch of risk classifications between vector and disease indicates that the prevention of malaria resurgence should not over-rely on routine surveillance of local *Anopheles*.

Risk classification could be considered in malaria prevention. Lingala et al. proposed that classified rainfall could help provide early warning of impending malaria outbreaks.^67^ Stoler et al. also classified the distance for the effect of urban agriculture on self-reported malaria in Ghana.^68^ In addition to assessing the relationship between environmental variables and malaria transmission, classification helps control the outbreak risk of *An. sinensis* and malaria transmission. Harvey et al. developed the first malaria epidemic early warning system and using classifications that meet the conditions.^69^ They further tested this system and discovered that for the high alert threshold, precision increased to > 99% and recall to 5%. In fact, several species, including the *Oncomelania hupensis*,* Aedes albopictus*, and *Culicoides*, have successfully used the output predictions from similar models for risk classification, which could contribute to the development of effective strategies to prevent further spread.^47^^,^^49^^,^^70^ Similarly, the risk classification was based on the values of the output predictions from the ensemble models in our study. A high-risk class indicates high environmental suitability of the species in this area. By identifying where the risk class is high, we can determine the hotspots for the species in an area, which could provide early warning of *An. sinensis* and malaria transmission. It did not ensure that *An. sinensis* or malaria transmission occurs in high-risk or risk areas but rather provided possible specific guidance on priorities for the prevention and control of *An. sinensis* and malaria transmission. Our classification maps indicate some high-risk areas where prevention and control could be strengthened.

The risk distributions of malaria transmission and presence of *An. sinensis* were influenced by multiple factors. IM and AAT greatly influenced the risk distribution for *An. sinensis* in ensemble models, which is consistent with results from previous studies.^71^^,^^72^ Host factors such as the NLI and Human Footprint strongly influenced the risk distribution for malaria transmission in ensemble models. With the expansion of the population in Shanghai due to economic development and urban expansion, the growth in susceptible populations will further lay the groundwork for the resurgence of malaria.

The ensemble model performed best in our study. For a single model, the out-of-sample performance of RF and FDA was best, and SRE and MAXENT were worst. Although MAXENT has been used in some studies to predict malaria distribution,^18^^,^^39^^,^^73^ its out-of-sample performance was less ideal in our study. Further, studies have shown that a single model did not perform well under various conditions,^35^^,^^46^^,^^74^^,^^75^ which influences the effectiveness of model predictions.^44^^,^^76^ Indeed, the results of the application of the traditional method that selected the best from multiple models often differ from actual observations, especially when the observation set is spatially or temporally independent from the calibration set.^77^^,^^78^ In our study, the ensemble modeling technique was applied to deal with intermodel uncertainty^52^^,^^79^ and was more robust and accurate in prediction.

There are some limitations in our study. The model used geographic locations but did not consider *An. sinensis* density, which plays a crucial role in disease transmission.^49^ Further, the *An. sinensis* locations from literature differed in sampling and resolution, where spatial autocorrelation might exist. For this, we supplemented occurrences for *An. sinensis* found in the normative field surveillance and occurrences in the GBIF database to the raw database. Although not all environmental variables were available for the 2010–2020 period with enough resolution, we tried to use stable variables that cover or approach this period and interpolation analysis to improve the resolution of the raster layer created by the ensemble models for *An. sinensis* and malaria transmission. Also, fuzzy kappa was used to measure the differences caused by interpolation per grid statistically. However, temporal effects caused by different years of occurrence and variables might be limitations for this kind of study for predicting risk distribution by variables, which has been identified in previous studies.^32^^,^^47^ Finally, only locations, where the malaria cases were located at the time of diagnosis, were included in our study. Theoretically, all locations experienced by a patient during the infectious period are at spatial risk of transmission at the same time and should be considered.^73^^,^^80^

In conclusion, we established ensemble models combining multiple algorithms that better predicted the risk distributions for *An. sinensis* and malaria transmission in Shanghai. Environmental predictors represented by climate and host factors indicated strong importance among different model algorithms. In Shanghai, risk areas for *An. sinensis* and malaria transmission varied in size and level in the 16 districts, but there was not a strong relationship between *An. sinensis* and malaria risk classification. Beyond precise *An. sinensis* monitoring in transmission risk areas of malaria, the challenge of malaria in Shanghai asks for the focus of malaria surveillance even in low-risk areas for *An. sinensis*.

## Supplemental files


Supplemental materials
