# Supplementary figures and images for: Prediction of the Risk Distributions for Anopheles sinensis, a Vector for Malaria in Shanghai, China
Source: Am J Trop Med Hyg. 2023 Jan 23;108(3):599–608. doi: 10.4269/ajtmh.22-0523 (PMC9978570; doi:10.4269/ajtmh.22-0523)

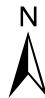

Fuzzy Kappa of  
*Anopheles sinensis*

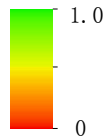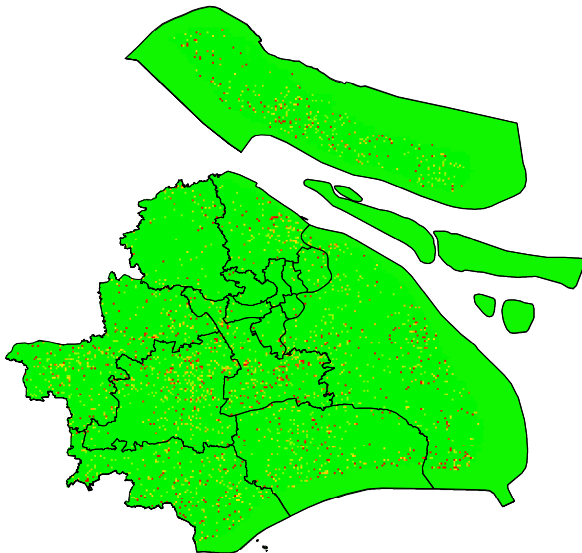

Fuzzy Kappa of  
malaria transmission

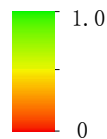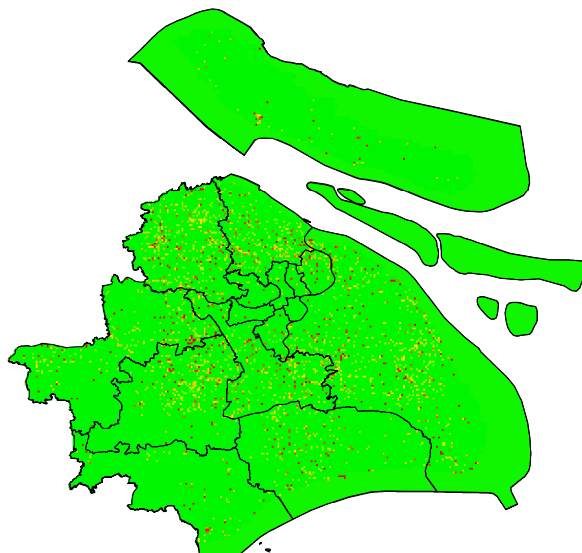

0 10 20 40 60 km

Supplement: Supplementary file 1 [file tpmd220523.SD1.pdf]
